# Practical Guidance for Outpatient Spasticity Management During the Coronavirus (COVID-19) Pandemic: Canadian Spasticity COVID-19 Task Force

**DOI:** 10.1017/cjn.2020.104

**Published:** 2020-05-26

**Authors:** Rajiv Reebye, Heather Finlayson, Curtis May, Lalith Satkunam, Theodore Wein, Thomas Miller, Chris Boulias, Colleen O’Connell, Anibal Bohorquez, Sean Dukelow, Karen Ethans, Farooq Ismail, Waill Khalil, Omar Khan, Philippe Lagnau, Stephen McNeil, Patricia Mills, Geneviève Sirois, Paul Winston

**Affiliations:** GF Strong Rehabilitation Centre, Vancouver, British Columbia, Canada; Division of Physical Medicine and Rehabilitation, University of British Columbia, Vancouver, British Columbia, Canada; Faculty of Medicine, University of British Columbia, Vancouver, British Columbia, Canada; Glenrose Rehabilitation Hospital and Division of Physical Medicine & Rehabilitation, University of Alberta, Edmonton, Alberta, Canada; Stroke Prevention Clinic, Montreal General Hospital and McGill University Health Center, Montreal, Québec, Canada; St. Joseph’s Health Care London, Western University, London, Ontario, Canada; West Park Healthcare Centre, Toronto, Ontario, Canada; Physical Medicine and Rehabilitation, University of Toronto, Toronto, Ontario, Canada; Stan Cassidy Centre, Fredericton, New Brunswick, Canada; Division of Physical Medicine and Rehabilitation, Dalhousie University, Halifax, Nova Scotia, Canada; Department of Clinical Neurosciences, University of Calgary, Calgary, Alberta, Canada; Department of Internal Medicine’s Section of Physical Medicine and Rehabilitation, University of Manitoba, Winnipeg, Manitoba, Canada; Department of Physical Medicine and Rehabilitation, Saskatoon City Hospital, University of Saskatchewan, Saskatoon, Saskatchewan, Canada; Regional Rehabilitation Centre, Hamilton, Ontario, Canada; Hotel Dieu Shaver Health and Rehabilitation Centre, St. Catharines, Ontario, Canada; Foothills Medical Centre, Calgary and Department of Clinical Neurosciences, Alberta, Canada; University of Calgary, Calgary, Alberta, Canada; Institute of Rehabilitation and Physical Impairment of Quebec City and Laval University, Québec City, Québec, Canada

**Keywords:** Spasticity, Physical medicine and rehabilitation, COVID-19 pandemic, Guidance

## Background

Spasticity is a common sequela of upper motor neuron conditions that can reduce quality of life, impair function, and heighten economic burden.^1^ Identification and treatment of problematic spasticity is key in order to decrease impairments including contracture formation, pain, skin breakdown, and functional decline and to limit disability.^2^

The COVID-19 pandemic has affected health-care systems worldwide including physical medicine and rehabilitation (PMR) and neurology practices in Canada.^3-5^ Inpatient hospital care for the management of patients with COVID-19 has been prioritized, while elective surgeries and outpatient clinics have been limited or canceled as part of resource allocation management and mitigation of risks during the pandemic. Subsequently, the delivery of outpatient spasticity care in the PMR and neurology setting has been directly impacted.^6^

The great majority of Canadian spasticity outpatient clinics have been canceled or are now predominantly administered through telemedicine, which has led to a dramatic reduction in patient volume.

As the pandemic evolves, there is a possibility of continuous or intermittent physical distancing being required until a vaccine becomes available.^7^

For this reason, a COVID-19 spasticity task force was created. This task force is comprised of 17 Canadian experts in the field of spasticity management. These 16 PMR specialists and 1 neurologist have collaborated extensively regarding spasticity clinical practice, research, and education for more than 10 years nationally and internationally. Our task force experts have academic appointments from 10 Canadian universities and representation at the executive level in the Canadian Association of Physical Medicine and Rehabilitation, Canadian Advances in Neuro-Orthopedics for Spasticity Congress, and Canadian Stroke Best Practices Advisory Committee. There was an attempt for geographic diversity by seeking representation from all provinces with spasticity clinics, but we lacked representation from Nova Scotia, Prince Edward Island, and Newfoundland and the territories. We did not specifically attempt to attain gender diversity. Almost all spasticity clinics in Canada are run by physiatrists while only a very small number are headed by neurologists, hence the majority of task force members are physiatrists.

The COVID-19 spasticity task force conducted their first web-based meeting on April 21, 2020, during which consensus was obtained based on expert opinion for strategies to optimize spasticity outpatient care during the pandemic. Subsequent web-based, telephone, and email discussions regarding regional approaches to the response and procedures of spasticity clinics during the pandemic were conducted and the conclusions are included in our guidance paper. Our task force recommends that clinicians continue to deliver spasticity management via telemedicine for both follow-up care and new patient assessments during this pandemic.

We have also identified a subset of patients that require in-person assessment and access to treatment modalities such as intrathecal baclofen pump therapy, focal chemodenervation, and orthotic adjustments.

In this practical guidance paper, we outline a triaging strategy to determine the urgency of managing spasticity patients in-person versus using telemedicine. We provide strategies to best protect patients, physicians, caregivers, and staff from possible exposure to COVID-19 during outpatient visits. We acknowledge that there may be significant regional differences in personal protective equipment (PPE) access and restrictions regarding provision of care for outpatient clinics. Practitioners should refer to their local and hospital guidelines to ensure that they are in compliance with current recommendations, and our suggestions should be adapted accordingly.

## Patient Selection for Telemedicine versus In-Person Assessments

Spasticity clinics should not be canceled. Our opinion is that the majority of our spasticity patients should be assessed by telemedicine as a temporary measure. This includes patients who are treated without interventional procedures, those who are treated with oral antispasmodics and/or were recently injected with botulinum toxin or phenol, and those who are stable on intrathecal baclofen treatment.

Telemedicine can be used to gather a history, assess function, perform basic physical exam evaluation, and establish and monitor treatment goals.^8^ It can also be used to help with stretching, range of motion, and spasticity education prior to in-person assessment. Telemedicine is obviously limited due to the inability to perform a full physical examination to adequately assess spasticity, tone, and contracture, and in certain clinical scenarios, in-person assessment becomes essential to ensure appropriate treatment.

## Triaging Strategy for In-Person Assessment

We estimate that approximately 10%–30% of spasticity patients will require an in-person assessment. It is important to carefully triage this group to determine the urgency for spasticity assessments in order to reduce unnecessary exposure to COVID-19, to conserve PPE, and to help decrease medical complications and emergency visits.

**Figure 1:**
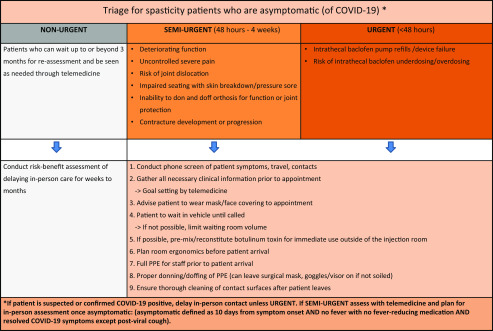
Practical guidance for outpatient spasticity management flowchart.

We propose the following patient triage strategy based on tiers of urgency (see Figure 1):

- **The URGENT GROUP** classified as a need for in-person assessment/intervention within 48 hours of contacting the clinic/physician with clinical scenarios including
  ○ patients requiring intrathecal baclofen pump refills to avoid withdrawal;
  ○ patients experiencing symptoms or signs of intrathecal baclofen underdosing or overdosing;
  ○ patients experiencing symptoms or signs of baclofen pump device failure.
- **The SEMI-URGENT GROUP** classified as a need for in-person assessment between 48 hours and 4 weeks and includes clinical scenarios where spasticity is worsening over the last 4 weeks and is associated with
  ○ deteriorating functions such as mobility (including falls and difficulty with transfers), perineal care/hygiene with increased caregiver needs, and/or inability to function independently;
  ○ increasing severe pain that is uncontrolled with other measures and has previously responded to chemodenervation;
  ○ increased risk of joint dislocation;
  ○ development of new or worsening pressure ulcer or wound;
  ○ inability to don and doff orthoses required for function;
  ○ impaired seating with increased pain and/or high risk of skin breakdown;
  ○ loss of joint range of motion affecting function, skin breakdown, and/or contracture development.
- **The NON-URGENT GROUP** classified as patients who can wait up to or beyond 3 months for reassessment and be seen as needed through telemedicine.

**For patients who are asymptomatic (of COVID-19) and in the urgent or semi-urgent groups:**

- Assume that asymptomatic transmission of COVID-19 is possible, and therefore use appropriate PPE for all in-person encounters to protect both patients and health-care providers.
- The WHO guidelines for use of PPE advise using surgical mask, goggles/face-shield/visor, gloves, and gown when treating/injecting patients in the outpatient setting.^9^

**For patients in the urgent group who are suspected or confirmed COVID-19 positive:**

- Urgent in-person assessment and treatment must be undertaken if there are intrathecal pump delivery system concerns or intrathecal baclofen refills needed.
- Strict PPE and safety precautions adhering to local guidelines on how COVID-19 positive patients are to be managed need to be in place.

**For patients in the semi-urgent group who are suspected or confirmed COVID-19 positive:**

- Assess with telemedicine and plan for in-person assessment once asymptomatic (defined as 10 days from symptom onset AND no fever without fever-reducing medication AND symptom resolution with the exception of post-viral cough).^4,10^
- In the scenario where in-person treatment is required, ensure strict PPE and safety precautions adhering to local guidelines on how COVID-19 positive patients are to be managed.

### Setup of Outpatient Clinics During Pandemic

- **Patient screening**
  ○ Screen patients by phone prior to in-person assessments to ensure no COVID-19 symptoms, including fever, cough, shortness of breath, chills, muscle pain, new loss of taste or smell, vomiting or diarrhea, and/or sore throat or close contacts who are ill prior to attending.^11^ Advise patients to use the local health authority self-assessment tool.
  ○ Ask if any family members are ill or COVID-19 positive or if they have had recent travel or exposure to a person who is COVID-19 positive; if so, delay the in-person clinic visit, offer telemedicine visit, and reassess in a timely fashion.
  ○ Given the current outbreaks in long-term care (LTC) facilities, we recommend not to transfer patients from LTC or other assisted living and group home settings to outpatient clinics during the pandemic unless urgent or semi-urgent. Telemedicine can be used to triage these patients into whether they may require urgent or semi-urgent in-person assessment. At the time of this publication, patients in LTC settings are not allowed to be transferred for non-urgent outpatient appointments in many regions of Canada.
  ○ Assess the need for an overhead lift to transfer a patient. Consider options to prepare the patient to be treated in their wheelchair if possible, such as loose clothing and tilt in space wheelchair.
  ○ Staff should acquire all patient information by telephone and limit interactions for information with patients during the in-person visit.
  ○ Arrangements for all follow-up appointments should be made by telephone to limit face-to-face interaction with front office staff.
- **Staff education regarding PPE and safety protocols**
  ○ Bring clean work clothes/scrubs/shoes for the clinic and another set of clothes for home.
  ○ Prior to leaving work or once home, shower and change into another set of clothing to decrease risk of transmission to family members/others living together.
  ○ Hand washing with soap and water or disinfecting gel is essential before and after every patient contact.
  ○ Ensure surgical masks, gloves, goggles/face visors fit properly before in-person contact with patients. Goggles/face visors (should be cleaned after each patient encounter) and masks can be reused for the day if not soiled or damaged.
  ○ Wear a gown if available (ideally water-resistant) on top of scrubs or clothing when in contact with patients.
  ○ N95 masks and face visors are recommended for high-risk encounters (i.e., aerosol generating procedures for patients).^9^
  ○ Appropriately dispose of gloves and gown after each procedure.
  ○ When seeing patients with dysphagia and/or facial weakness use a full-face visor due to the potential for increased droplet risk.
- **Setup of outpatient office and treatment room**
  ○ Create markings for physical distancing (6 feet or 2 meters separating front office staff from patients).
  ○ Avoid patients waiting in waiting room
    ▪ Ask the patient to wait in their vehicle, if possible, to minimize patients in the waiting area until the treatment room is adequately clean.
  ○ Dedicate a room as a clean room for staff to change and keep personal items.
  ○ Dedicate a room for assessment and/or injection, removing all non-essential objects/books/personal effects from room.
  ○ Space out each assessment/injection by at least 30 minutes to ensure adequate time for proper disinfection of rooms. Consider alternating appointments with telemedicine visits between in-person assessments.
  ○ Clean all patient contact points (e.g., chairs, doorknobs, ultrasound probes, electromyography (EMG) equipment, and lift system slings) with CAVI wipes ^TM^, isopropyl alcohol or a similar hard-surface disinfectant.
  ○ Dedicate, if possible, another room for dictation and paperwork.
  ○ Keep a separate injection supply kit/bag for each patient. Supplies can be kept in individual sealable plastic bags (with syringe, swab, alcohol swab) then disposed of after each patient encounter.
  ○ If possible, pre-mix/reconstitute botulinum toxin for immediate use outside of the injection room. This process can often be facilitated by preloading syringes with the appropriate amount of saline immediately before the clinic to streamline the reconstitution process once the patient arrives.
  ○ Position bed, ultrasound, EMG, and/or other equipment in an ergonomically optimized orientation prior to the patient entering the room.
  ○ No more than two staff should be in the room if possible (nurse and physician) when assessing/injecting the patient.
  ○ Limit the number of physicians running spasticity clinics at the same time in the same area.
  ○ When trainees are present, all precautions should be strictly adhered to and bedside teaching can occur outside the treatment room.
- **Patient education**
  ○ Before arrival, explain the changes in place in the clinic during the pandemic and reinforce the need for physical distancing.
  ○ Advise the patient to bring a mask, which may be homemade, or provide a mask upon arrival to the clinic.
  ○ Advise no visitors or family members during the consultation/procedure unless essential (e.g., requiring support as a result of cognitive/language dysfunction).
  ○ Advise not to bring purses/bags/unnecessary items to the clinic.
  ○ Advise patients to wear clothing that is easily removable or accessible to reduce preparation time and potentially avoid the need for the use of an overhead lift to expose targeted regions.

## Conclusion

It is essential to provide timely spasticity management while protecting patients and multidisciplinary spasticity teams from COVID-19 exposure.

Telemedicine is an effective tool for triage, assessment, and teaching for the majority of patients. However, there will be patients that require in-person contact for spasticity management procedures in order to maintain function and reduce the risk of significant complications and emergency department visits during this pandemic.

Triaging patients into non-urgent versus semi-urgent or urgent groups is essential to determine the need for in-person assessment. Patient education regarding changes in the delivery of spasticity care during the pandemic, staff education regarding PPE, triage protocols, room setup for examination and injection procedures, and outpatient clinic logistics are key in successfully treating spasticity during this pandemic. It is crucial to remember that our purpose is to maximize targeted and appropriate care to all patients during this unprecedented time and as spasticity specialists we will strive to safely provide the best possible care.
